# Familial Mediterranean fever in Romania: a case report and literature review

**DOI:** 10.3389/fped.2024.1546387

**Published:** 2025-01-15

**Authors:** Alin Iuhas, Cristian Marinău, Larisa Niulaș, Zsolt Futaki, Andreea Balmoș, Kinga Kozma, Mirela Indrieș, Cristian Sava

**Affiliations:** ^1^ Faculty of Medicine and Pharmacy, University of Oradea, Oradea, Romania; ^2^ Bihor County Clinical Emergency Hospital, Oradea, Romania

**Keywords:** familial Mediterranean fever, Romania, autoinflammatory disease, recurrent fever, FMF

## Abstract

Familial Mediterranean Fever (FMF) is a hereditary autoinflammatory disease characterized by recurrent fever and systemic inflammation, most prevalent in Eastern Mediterranean populations. Rare in regions like Romania, FMF presents diagnostic challenges and risks severe complications if untreated. We report a 7-year-old Romanian girl, from a non-classical ethnic background, with recurrent febrile episodes and elevated inflammatory markers. Genetic testing confirmed a homozygous *MEFV* c.2082G>A (p.Met694Ile) variant. Colchicine therapy reduced flare frequency and normalized inflammatory markers. FMF should be considered in atypical populations with recurrent inflammation. Genetic testing aids diagnosis in non-endemic regions, enabling early colchicine treatment to prevent complications.

## 1 Introduction

Familial Mediterranean Fever (FMF) is the most common and one of the earliest recognized autoinflammatory diseases. This hereditary condition is marked by recurrent episodes of fever and polyserositis, chronic inflammation which can lead to significant long-term complications, including renal amyloidosis (1).

FMF is predominantly found in Eastern Mediterranean populations, such as non-Ashkenazi Jews, Armenians, Turks, and Arabs (2). The highest prevalence of FMF is found in Turkey, with a reported rate of 1 in 1,000. However, there are significant regional differences, with the northwestern region of Turkey showing a prevalence as low as 6 in 10,000 (2, 3). In Italy, the distribution of FMF cases also differs significantly between northern and southern regions, with the southern districts exhibiting a much higher prevalence of the disease, explained by ancient colonization of the region by Greeks and Arabs, as well as the migration patterns of Jewish populations (1, 4).

*MEFV* gene (from MEditerranean FeVer) encodes a 781 amino acid protein called Pyrin. Pathogenic variant of the gene cause a loss of function in this protein which plays a role in maintaining homeostasis (5).

The clinical presentation of FMF can vary, influenced by genetic diversity and environmental factors, but it typically features recurrent fever and systemic inflammation, including serositis. Beginning in childhood, patients experience brief, self-limiting episodes of fever accompanied by abdominal, chest, or joint pain and systemic inflammation (6).

We report a case of recurrent fever in a child from a Romanian family, with no family history of familial mediterranean fever and no typical ethnic background associated with the condition, in a geographical area where this pathology is rare, and the diagnosis is often delayed, sometimes only being established after complications of the disease have already developed (7).

## 2 Case report

We present the case of a 7-year-old female patient who has been under observation since the age of 5 in the Pediatric Department of Bihor County Emergency Hospital. The patient's medical history is notable primarily for caloric-induced obesity, with no other significant personal or family medical issues reported. Her medical and developmental history otherwise remains unremarkable, with normal growth and age-appropriate milestones, and no chronic illnesses.

The patient initially presented at the age of 5 with urinary discomfort, fever, and pain in the left hypochondrium. Ultrasound revealed mild splenomegaly and the presence of an accessory spleen. Laboratory tests showed leukocyturia, elevated CRP (161 mg/L), and ESR of 25 mm/h. A urinary tract infection was suspected despite the negative urine culture, and antibiotic treatment was initiated, ultimately leading to a favorable outcome.

**Figure 1:**
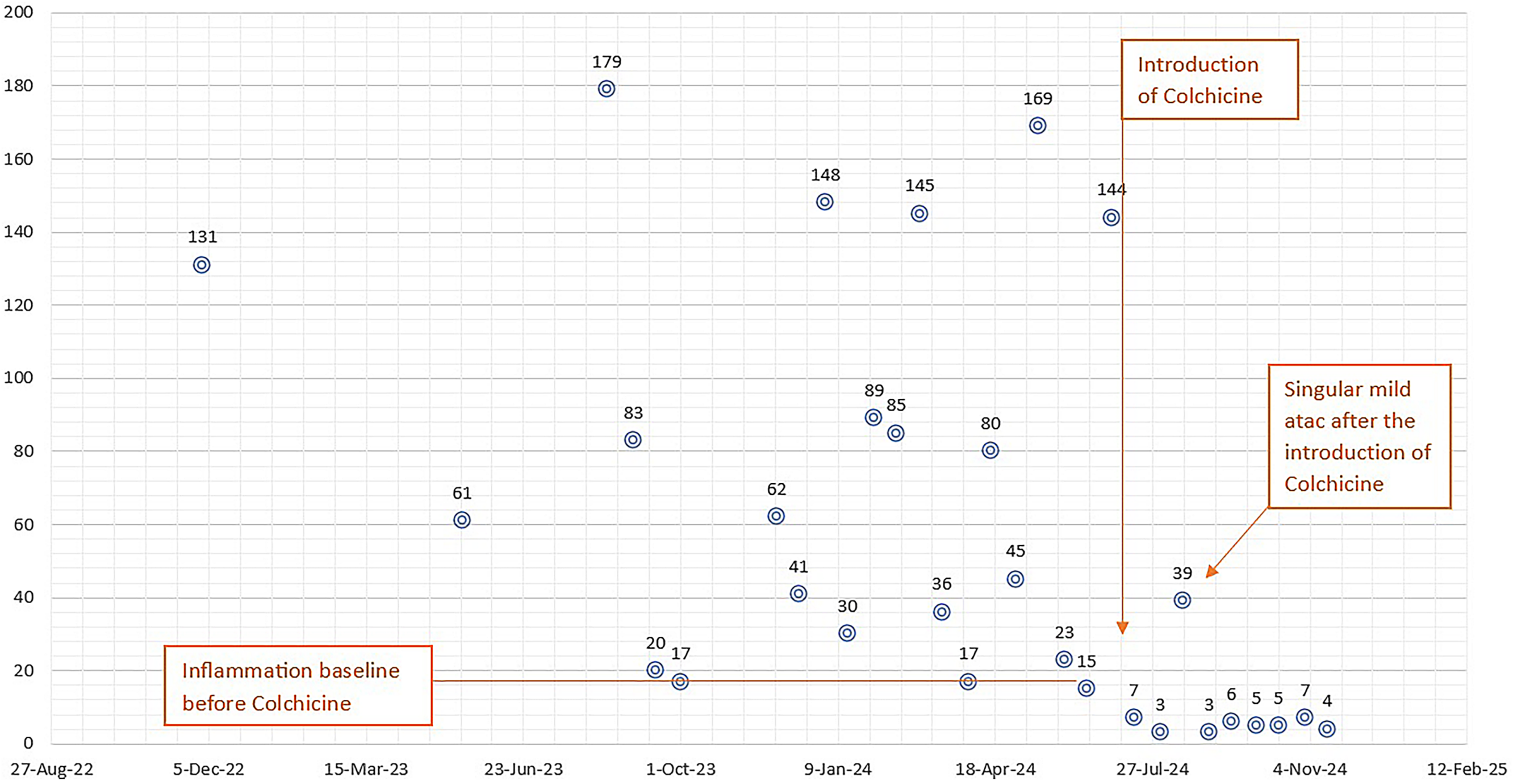
The frequency of inflammatory attacks and C-reactive protein (CRP) levels (in mg/L on the vertical axis) over the 2-year observation period (horizontal axis) are shown; it can be observed that after the introduction of colchicine the baseline inflammation, initially around 20 mg/L, normalizes, and there is a significant reduction in inflammation during an attack.

Over the following year, she experienced several recurring episodes, initially spaced approximately 1–2 months apart (Figure 1). Throughout this time, her clinical manifestation were dominated by recurring episodes of fever and abdominal pain. Additionally, she experienced an autolimited episode of bilateral shoulder arthralgia. The frequency of her inflammatory episodes increased over time, occurring bimonthly with notable elevations in inflammatory markers, such as CRP levels fluctuating from 80 to 180 mg/L, with baseline levels at 20–30 mg/L between episodes. Repeated cultures were performed, along with serology for Borrelia, EBV, CMV, Parvovirus B19, SARS-CoV-2, HIV, hepatitis B and C, immunogram, ANA, HLA-B27, liver and kidney function tests, multiple peripheral blood smears, all of which were within normal limits. The abdominal CT, which confirmed the splenomegaly, was otherwise unremarkable.

Approximately 12 months into this pattern of recurring and autolimited episodes of inflammation with abdominal pain, an autoinflammatory disease was suspected. The patient meets 6 out of the 9 Eurofever/PRINTO criteria for suspected FMF, specifically: the presence of inflammatory attacks lasting typically 1–2 days, abdominal pain, and the absence of aphthous stomatitis, urticarial or maculopapular rash, and lymphadenopathy. A comprehensive genetic panel for inflammatory diseases was considered appropriate to aid in reaching a diagnosis and Colchicine therapy was initiated at 1 mg per day to better control her symptoms. The results of the genetic test show a homozygous mutation in the *MEFV* gene at position c.2082G>A, corresponding to the p.Met694Ile variant (M694I), thus confirming a diagnosis of Familial Mediterranean Fever.

Since starting colchicine, she has demonstrated good gastrointestinal tolerance and an overall favorable clinical course. In the 6-month period following the initiation of colchicine therapy, she has experienced only one mild flare, marked by mild pain without fever and a moderate elevation in CRP to 60 mg/L. Between these episodes, her acute-phase reactants have remained within normal limits, and serum amyloid A levels was in normal range.

## 3 Discussions and literature review

This case highlights the challenge of diagnosing FMF in a geographical area where this pathology is rare.

FMF has its highest prevalence in the eastern Mediterranean region, with Turkey showing the greatest concentration of cases today. Over 70% of cases in Turkey are found in central and eastern Anatolia as well as the inner Black Sea regions. The incidence of FMF decreases in proportion to the distance from this epicenter, reflecting historical migration routes of populations with a genetic predisposition to the disease, including Turks, Greeks, Arabs, and Jews. Turkey reports an average prevalence of FMF of approximately 1 in 1,000, though there is considerable interregional variability. In Israel, the prevalence is estimated to be around 1–2 cases per 1,000 individuals, a rate similarly observed in the Armenian population (2–4). FMF is also found in North African countries, Greece, Crete, France, Germany, and the United States. In most of these regions, the occurrence of FMF is primarily linked to significant immigration from Mediterranean countries. Milder forms of the disease have been observed in patients who migrated to these countries, regardless of their ethnicity, while patients from regions like Turkey and Armenia show a higher predisposition to developing renal amyloidosis. This increased risk has been attributed to the role of infections as a trigger for inflammatory attacks (8). In Eastern and Central European countries, including Romania, the estimated prevalence of FMF is significantly lower. A multinational study conducted in 2010 estimated the prevalence to be approximately 1 in 465,500 children between the ages of 0 and 19. This figure includes both genetically confirmed cases and suspected cases of FMF, with genetically confirmed cases accounting for about 18% of the total (9).

The *MEFV* gene consists of 10 exons and is located on chromosome 16p13.3. To date, more than 400 variants have been identified and documented in the INFEVERS database (Infevers, Sarrauste de Menthiere et al. https://infevers.umai-montpellier.fr, accessed in 04.11.2024) However, for the majority of these variants, pathogenicity or frequency cannot yet be clearly established; only 6 of them have been classified as pathogenic, with an additional 58 classified as likely pathogenic. The primary hot spots for FMF- causing *MEFV* variants have been identified on exon 2, at position 148, and on exon 10, at positions 680 and 694 (10). The variant identified in our patient, *MEFV* c.2082G>A, p.(Met694Ile), is located on exon 10. In its homozygous form, this variant is known to be pathogenic and is believed to have originated in Turkey. The alteration of the methionine residue at position 694 was identified as a high-penetrance mutation when the pyrin gene was first discovered in 1997. Furthermore, although FMF is typically considered an autosomal recessive disease, cases have been reported where patients with the classic FMF phenotype exhibited what appeared to be a dominant pattern of transmission (11). It has been observed that a more severe phenotype, characterized by high fever, and musculoskeletal manifestations, is usually associated with high penetrance mutations (12). One example is M694V, which appears to be associated with a less favorable response to colchicine (13). On the other hand, a mild phenotype or incomplete penetrance has also been reported in patients with K695R or P369S variants (14).

The *MEFV* gene encodes a protein consisting of 781 amino acids, known as pyrin, which exists in multiple isoforms located in both the cytoplasm and nucleus. When activated, pyrin oligomerizes with other cellular proteins to form a macromolecular structure called the “pyrin inflammasome”. This inflammasome subsequently activates caspase-1, which facilitates the release of the pro-inflammatory cytokine IL-1β (15).

FMF is characterized by recurring, short-lived inflammatory episodes that typically resolve on their own within 1–3 days. The classic, hallmark symptoms of FMF include fever, serositis, and erysipelas-like erythema (16). Some FMF patients may develop disease-related complications over time, while others may initially present with them. A typical example of the latter is amyloid A (AA) amyloidosis occurring in the absence of classical FMF symptoms (15, 17). Our patient comes from a rural background, living at a significant distance from the nearest medical center, and her family's socio-economic situation is modest. Self-medication as a first response, often administering antibiotics at home before seeking medical care, combined with generally low compliance with prescribed treatment and delayed hospital presentations, sometimes occurring days or even weeks after the onset of inflammatory attacks, has contributed to a delayed diagnosis. At these presentations, cultures collected have consistently returned negative results, complicating the assessment of her inflammatory episodes; it remains unclear whether these episodes are genuinely non-infectious in nature, as is typical of FMF, or if the negative cultures reflect prior antibiotic administration at home. In hindsight, even the initial hospital presentation was an FMF attack, but at that time, the symptoms mimicked a pyelonephritis and the patient was treated accordingly, despite the negative urine culture, which was collected under suboptimal conditions (sample taken without rigorous local hygiene and while on antibiotic treatment).

Several triggers for FMF attacks have been identified, including infections, stressful events, cold exposure, and the menstrual cycle in pubertal and post-pubertal females. Approximately 50% of FMF patients experience a prodrome, often presenting as a general sense of malaise and discomfort (18–20). In our patient's case, most attacks occurred in the context of infections, where specific treatment for the infection only partially improved her clinical condition. During the cold season, with the increased incidence of respiratory infections in the pediatric population, her attacks became bimonthly. Although conclusive data is lacking, diet appears to play a role in triggering the disease, with some authors suggesting that salty or fat-rich foods may be a contributing factor. Additionally, correlations have been found between wheat consumption and the onset of attacks. A diet rich in antioxidants and supplements with anti-inflammatory effects could potentially reduce symptoms and improve the well-being of FMF patients (21).

FMF diagnosis is based on patient history, inflammatory markers, and increasingly on genetic testing. Various diagnostic criteria sets, including the Tel Hashomer, Livneh, and Turkish pediatric criteria, focus on clinical symptoms, family history, and response to colchicine. The Tel Hashomer criteria, the oldest and most widely used, define typical attacks by recurrent pain, fever (≥38°C, rectal temperature), short duration (12 h–3 days), and at least one of the following: severe peritonitis, unilateral pleuritis, pericarditis, monoarthritis (knee, ankle, or hip), erysipelas-like rash, or symmetric myalgia; several minor criteria and additional indicators were recognized (positive therapeutic response to colchicine, family medical history suggestive of FMF, ethnic background consistent with FMF prevalence, parental consanguinity and others) (16, 22). In 2009, a study observed that these criteria, although highly successful in identifying patients (sensitivity of 98.8%), have low specificity in the pediatric population (54.6%). The same study noted that the intensity and duration of symptoms, as described by the Tel Hashomer criteria, are reduced in the pediatric population, and some minor or supportive criteria cannot be applied. The Turkish pediatric criteria for FMF were established, incorporating family history and clinical features such as fever, arthritis, abdominal pain, and chest pain. A diagnosis of FMF can be made in children with two or more of these criteria, with a reported sensitivity of 86.5% and specificity of 93.6% (Table 1) (22). The evidence-based Eurofever/PRINTO classification criteria, developed in 2019 for inherited recurrent fevers, introduced for the first time an association between clinical and genetic variables, resulting in a high sensitivity and high specificity diagnosis; they also developed second set of criteria based solely on clinical criteria, as a possible tool for the indication for molecular analysis (Table 1) (23).

**Table 1:** Diagnostics criteria for familial Mediterranean fever.

Yalcinkaya-Ozen criteria (22)	Eurofever/PRINTO criteria (23)			
	Patients with molecular test		Patients without molecular test	
	Presence of confirmatory *MEFV* genotype and at least one among the following:	Presence of not confirmatory *MEFV* genotype (variant of uncertain significance) and at least two among the following:	At least six out of nine for a suspicion of FMF and the indication for molecular analysis:	
• Fever (axillary temperature of >38.8C, min. 3 attacs)	• Duration of episodes 1–3 days	• Duration of episodes 1–3 days	Presence	Absence
• Abdominal pain (6–72 h of duration, min. 3 attacs)	• Arthritis	• Arthritis	• Eastern Mediterranean ethnicity	• Aphthous stomatitis
• Chest pain (6–72 h of duration, min. 3 attacs)	• Chest pain	• Chest pain	• Duration of episodes, 1–3 days	• Urticarial rash
• Arthritis (6–72 h of duration, min. 3 attacs)	• Abdominal pain	• Abdominal pain	• Chest pain	• Maculopapular rash
• Family history of FMF			• Abdominal pain	• Painful lymph nodes
			• Arthritis	
Note: the presence of two or more of these five criteria diagnosed FMF with a sensitivity of 86.5% and a specificity of 93.6%	Note: these criteria confers 94% sensitivity and 95% specificity		Note: these criteria confers 91% sensitivity and 92% specificity	

A recent study conducted on the largest childhood FMF cohort in the United States, a non-endemic area, concluded that a negative family history should not exclude FMF as a potential cause of periodic fever. In some cases, recurrent fever may be the only symptom, particularly in young FMF patients (24).

The differential diagnosis during the acute phase includes infectious diseases, abdominal surgical pathologies, and malignancies (25, 26). However, once the self-limiting and recurrent nature of symptoms is established, conditions such as immunodeficiencies, cyclic neutropenia, inflammatory bowel diseases, multisystem inflammatory syndrome in children (MIS-C) and other autoinflammatory diseases (AIDs)—primarily periodic fever, aphthous stomatitis, pharyngitis, adenitis (PFAPA) syndrome, mevalonate kinase deficiency (MKD), TNF receptor-associated periodic syndrome (TRAPS), and cryopyrin-associated periodic syndrome (CAPS), must be excluded (26–29). Some patients develop FMF in association with other autoinflammatory diseases, experiencing more severe forms of illness than those with FMF alone. In some cases, these associated conditions are diagnosed before FMF. In large pediatric FMF cohorts, the most common co-existing diseases were Henoch–Schoenlein purpura (HSP), Juvenile Idiopathic Arthritis (JIA) (particularly spondyloarthropathies), polyarteritis nodosa (PAN), Behçet's disease, inflammatory bowel disease, and multiple sclerosis. The risk of JIA, HSP, and PAN is estimated to increase by 24, 62, and 112 times, respectively, in FMF patients compared to the general population (26–28).

The objectives in treating FMF include enhancing quality of life, decreasing the frequency, severity, and duration of attacks, and preventing long-term complications, especially AA amyloidosis, by reducing chronic and subclinical inflammation (15, 30, 31).

The first-line treatment for familial mediterranean fever is colchicine. In addition to the indirect action on chemotaxis, motility, and stimulation of leukocytes, colchicine has been demonstrated to inhibit the NLRP3 inflammasome, thereby suppressing caspase-1 activation (32). The European League Against Rheumatism (EULAR) recommends the following initial colchicine dosages: for children under 5 years, up to 0.5 mg/day; for ages 5–10 years, 0.5–1.0 mg/day; and for those over 10 years and adults, 1.0–1.5 mg/day. If inflammation persists despite the initial colchicine dose, it can be increased by 0.5 mg/day, with a maximum of 2 mg for children and 3 mg for adults, monitoring for side effects. Colchicine should not reach doses of 0.1–0.2 mg/kg, as these are toxic or lethal (26). Lifetime colchicine prophylaxis is recommended for all patients, regardless of symptoms, unless a severe side effect occurs. In patients who consistently adhere to colchicine therapy, the long-term risk of amyloidosis is less than 1%, even if attacks are not completely controlled (15). Colchicine is a safe medication that has been used for many years [even during pregnancy (33)]; however, it has a narrow therapeutic index, and its most common side effects can occur even at therapeutic doses. These side effects are primarily gastrointestinal and include cramping, abdominal pain, hyperperistalsis, diarrhea, and vomiting. These symptoms occur in 10%–15% of patients and typically resolve after a period of treatment or with a dose reduction (34). Despite optimal treatment, approximately 5% of patients do not respond to the maximum tolerated dose of colchicine, while 20%–40% have an incomplete response, resulting in a reduction of fever episodes but not complete control (15). In 2016, EULAR defined resistance to colchicine as experiencing minimum one attack per month in compliant patients receiving the maximum tolerated dose for at least six months (35).

Additional treatment is available for patients with colchicine resistance or incomplete response. Inhibition of pyrin, which is involved in the synthesis of IL-1, offers a new approach for the treatment of FMF (31, 36). The three types of anti-IL-1 treatments are Anakinra, a human recombinant analogue of the IL-1 receptor antagonist (IL-1Ra); Rilonacept, a fusion protein that contains the extracellular domain of the type I IL-1 receptor fused with the Fc portion of IgG1; and Canakinumab, a fully humanized monoclonal antibody of the IgG1 class that targets IL-1 beta (37, 38). In recent years, substantial evidence has highlighted the important role of anakinra in preventing serositis attacks in patients with colchicine-resistant FMF (39, 40). The analysis of site-specific attacks, significant differences between the anakinra and placebo groups were observed only for joint attacks. In this context, anakinra may complement colchicine treatment, which is often effective in controlling activity in other locations but less so in preventing joint attacks. Additionally, several case studies have shown improvement in renal function in patients with amyloidosis following anakinra treatment (37). A 2012 study showed that rilonacept significantly reduced FMF attack frequency (41). A significant reduction in proteinuria was observed in FMF patients with amyloidosis who were treated with Canakinumab (42). All anti-IL-1 treatments carry a higher risk of infections, and injection site reactions are the most commonly reported side effect (37, 41, 42) However, gastrointestinal side effects are rare with anti-IL-1 biologics. Additionally, drug-drug interactions are unlikely, and their therapeutic window is not narrow, unlike that of colchicine. Moreover, studies indicate that there is no increased risk of any disease associated with long-term high-dose usage (43, 44).

FMF has an excellent prognosis if diagnosed and treated early, before complications arise. Serious complications typically occur only in patients with colchicine resistance or delayed diagnoses (45). Systemic AA amyloidosis is the leading cause of morbidity and mortality in FMF. After diagnosis, end-stage renal disease typically occurs within 5 years, with a 5-year survival rate of 50% (46). Recognizing the diverse challenges posed by the disease—such as physical symptoms, educational obstacles, and psychosocial impacts—can help healthcare providers, educators, and families design targeted interventions to improve the well-being of affected children. The Pediatric Quality of Life Inventory (PedsQL), a widely used tool for chronic illness monitoring, effectively assesses general health and disease-specific conditions by evaluating quality of life across physical, emotional, social, and academic domains. Research suggests that pediatric FMF patients often experience lower educational achievement and reduced quality of life (26).

With the increased availability and decreased costs of molecular tests, they have proven to be indispensable in diagnosing many pathologies (47, 48), including autoinflammatory diseases. Although rare in the geographical area of Romania, these diseases are still present, and diagnosing them before complications arise is essential. The nonspecific clinical presentation can pose challenges for clinicians who are not familiar with this pathology, making diagnosis difficult. Furthermore, in non-endemic areas, patients may present with a negative family history and few diagnostic criteria (23). In such cases, molecular tests are crucial for accurately establishing the diagnosis.
